# Estimated severe pneumococcal disease cases and deaths before and after pneumococcal conjugate vaccine introduction in children younger than 5 years of age in South Africa

**DOI:** 10.1371/journal.pone.0179905

**Published:** 2017-07-03

**Authors:** Claire von Mollendorf, Stefano Tempia, Anne von Gottberg, Susan Meiring, Vanessa Quan, Charles Feldman, Jeane Cloete, Shabir A. Madhi, Katherine L. O’Brien, Keith P. Klugman, Cynthia G. Whitney, Cheryl Cohen

**Affiliations:** 1 Centre for Respiratory Diseases and Meningitis, National Institute for Communicable Diseases, a division of the National Health Laboratory Service, Johannesburg, South Africa; 2 School of Public Health, Faculty of Health Sciences, University of the Witwatersrand, Johannesburg, South Africa; 3 Influenza Division, National Center for Immunization and Respiratory Diseases, Centers for Disease Control and Prevention, Atlanta, Georgia, United States of America; 4 Influenza Program, Centers for Disease Control and Prevention, Pretoria, South Africa; 5 School of Pathology, Faculty of Health Sciences, University of the Witwatersrand, Johannesburg, South Africa; 6 Division of Public Health Surveillance and Response, National Institute for Communicable Diseases of the National Health Laboratory Service, Johannesburg, South Africa; 7 Department of Internal Medicine, Charlotte Maxeke Johannesburg Academic Hospital, Johannesburg, South Africa; 8 Faculty of Health Sciences, University of the Witwatersrand, Johannesburg, South Africa; 9 Department of Paediatrics and Child Health, University of Pretoria, Steve Biko Academic Hospital, Pretoria, South Africa; 10 Medical Research Council: Respiratory and Meningeal Pathogens Research Unit, University of the Witwatersrand, Johannesburg, South Africa; 11 Johns Hopkins Bloomberg School of Public Health, International Vaccine Access Center, Department of International Health, Baltimore, Maryland, United States of America; 12 Hubert School of Public Health, Emory University, Atlanta, Georgia, United States of America; University of Otago, NEW ZEALAND

## Abstract

**Introduction:**

*Streptococcus pneumoniae* is a leading cause of severe bacterial infections globally. A full understanding of the impact of pneumococcal conjugate vaccine (PCV) on pneumococcal disease burden, following its introduction in 2009 in South Africa, can support national policy on PCV use and assist with policy decisions elsewhere.

**Methods:**

We developed a model to estimate the national burden of severe pneumococcal disease, i.e. disease requiring hospitalisation, pre- (2005–2008) and post-PCV introduction (2012–2013) in children aged 0–59 months in South Africa. We estimated case numbers for invasive pneumococcal disease using data from the national laboratory-based surveillance, adjusted for specimen-taking practices. We estimated non-bacteraemic pneumococcal pneumonia case numbers using vaccine probe study data. To estimate pneumococcal deaths, we applied observed case fatality ratios to estimated case numbers. Estimates were stratified by HIV status to account for the impact of PCV and HIV-related interventions. We assessed how different assumptions affected estimates using a sensitivity analysis. Bootstrapping created confidence intervals.

**Results:**

In the pre-vaccine era, a total of approximately 107,600 (95% confidence interval [CI] 83,000–140,000) cases of severe hospitalised pneumococcal disease were estimated to have occurred annually. Following PCV introduction and the improvement in HIV interventions, 41,800 (95% CI 28,000–50,000) severe pneumococcal disease cases were estimated in 2012–2013, a rate reduction of 1,277 cases per 100,000 child-years. Approximately 5000 (95% CI 3000–6000) pneumococcal-related annual deaths were estimated in the pre-vaccine period and 1,900 (95% CI 1000–2500) in 2012–2013, a mortality rate difference of 61 per 100,000 child-years.

**Conclusions:**

While a large number of hospitalisations and deaths due to pneumococcal disease still occur among children 0–59 months in South Africa, we found a large reduction in this estimate that is temporally associated with PCV introduction. In HIV-infected individuals the scale-up of other interventions, such as improvements in HIV care, may have also contributed to the declines in pneumococcal burden.

## Introduction

*Streptococcus pneumoniae* is a leading cause of bacterial pneumonia, meningitis, and sepsis, and is estimated to have caused approximately 335,000 (240,000–460,000) deaths in children aged <5 years in 2015 globally [1]. In 2008, before pneumococcal conjugate vaccines (PCVs) were available in low-income countries, the global estimated number of pneumococcal deaths was 541,000 (95% confidence interval [CI] 376,000–594,000) [2]. The method of estimation differed between the pre- and post-PCV global death estimates. With regards to risk groups in South Africa HIV-infected and HIV-exposed-uninfected children were shown to have a higher incidence of invasive pneumococcal disease (IPD) [3] and acute lower respiratory tract infections (LRTIs) compared to HIV-unexposed-uninfected children.[4].

Prevention of pneumococcal disease by PCVs has been documented through effectiveness and impact data from more than 50 countries [5]. In South Africa, the 7-valent pneumococcal conjugate vaccine (PCV7) was introduced nationally in April 2009 and replaced by PCV13 in June 2011. National surveillance data for invasive pneumococcal disease (IPD) from South Africa showed a 69% reduction in the incidence of all-serotype IPD among children aged <2 years by 2012, with contributions by PCV and HIV-associated interventions [6].

The burden of pneumococcal disease has been described in young children in the African region [7] with epidemiological studies in a number of countries including The Gambia [8, 9], Kenya [10] and South Africa [11]. World Health Organization (WHO) country specific estimates of disease burden [1, 7, 12] generate summed regional and global estimates using country-specific inputs for syndromic mortality (pneumonia and meningitis) along with other country-specific parameters (e.g. pathogen specific case fatality ratios, HIV prevalence, population size and vaccine coverage). South Africa has robust surveillance to measure IPD incidence and thus allows for an alternative model to estimate national cases and deaths from pneumococcal disease, anchoring on the observed value of IPD cases. The model can be updated over time to track improvements in health and be compared to other estimates.

We aimed to estimate the national burden of severe hospitalised pneumococcal disease and deaths (meningitis, bacteraemic and non-bacteraemic pneumonia, and non-pneumonia non-meningitis invasive disease) among HIV-infected and HIV-uninfected children aged 0–59 months in South Africa in two periods: 2005–2008, before PCV was introduced, and in 2012–2013, after PCV was introduced. Estimates were based on the observed IPD incidence measures from the national GERMS-SA surveillance system, as an alternative approach to that used by the WHO.

## Methods

### Data sources

#### GERMS-SA IPD surveillance programme

GERMS-SA is an active, national, laboratory-based surveillance programme for IPD and other invasive organisms. All public health sector microbiology laboratories (>200) are encouraged to submit isolates to the National Institute for Communicable Diseases (NICD) in Johannesburg. The public sector serves 84% of the South African population without private medical aid coverage [13]. Some private sector laboratories also submit isolates. Of the public sector facilities, 24 sites have dedicated surveillance officers who collect clinical information on identified patients thereby defining them as enhanced sites. Laboratory-based surveillance for IPD in South Africa began in 1999 [14]; the quality and strength of the surveillance programme was improved between 2003 and 2005. From 2005 the quality of the surveillance system was maintained with regular checks and audits [15]. Cases of IPD were defined as illnesses in patients with *S*. *pneumoniae* cultured from normally sterile-body sites (e.g. cerebrospinal fluid (CSF) or blood). Information on specimen type and age of cases is available from all sites; clinical diagnosis is reported only from cases that occurred at enhanced sites. Severe pneumococcal disease was considered as disease resulting in hospitalisation.

#### National Health Laboratory Service (NHLS) Corporate Data Warehouse (CDW)

The CDW is managed by the NHLS, the sole laboratory service provider for all public health facilities in South Africa. The CDW is a repository which contains archived data on all laboratory tests requested and results from public laboratories from 2003.

#### Additional input parameters for model

Values for input parameters of the model were derived from a number of sources. For estimates of non-bacteraemic pneumococcal pneumonia cases and adjustments for expected burden in presence of systematic blood culturing practices we used published data from a South African PCV9 vaccine clinical trial [16, 17]. Published data was also used to derive the CFR for non-bacteraemic pneumococcal pneumonia [18, 19].

#### Population denominators

Annual age-specific population denominators used to calculate incidence and mortality rates were obtained from Statistics South Africa [20]. The mid-year Statistics South Africa population estimates use the cohort-component method which is based on knowledge of population structure, birth rates, death rates and migration as well as assumptions of how they change. These estimates are derived from a census conducted in 2011 and are updated on an annual basis [20]. The Thembisa model, a demographic model designed to specifically address the population impact of changing HIV interventions [21], was used to estimate population denominators by HIV status; these denominators accounted for the changes in mother-to-child HIV transmission rates and improvements in paediatric antiretroviral (ARV) treatment. In this model the probability of mother-to-child transmission was assumed to depend on the mother’s HIV disease stage, the timing of ARV therapy initiation in pregnancy, the type of ARV prophylaxis received and for postnatal transmission the type of feeding. PCR testing in HIV-exposed infants was assumed to occur at birth and ARV treatment eligibility was expanded from all children aged <1 year to all children aged 1–4 years from mid-2012 based on updated South African ARV treatment guidelines [21].

### Model overview

**Fig 1:**
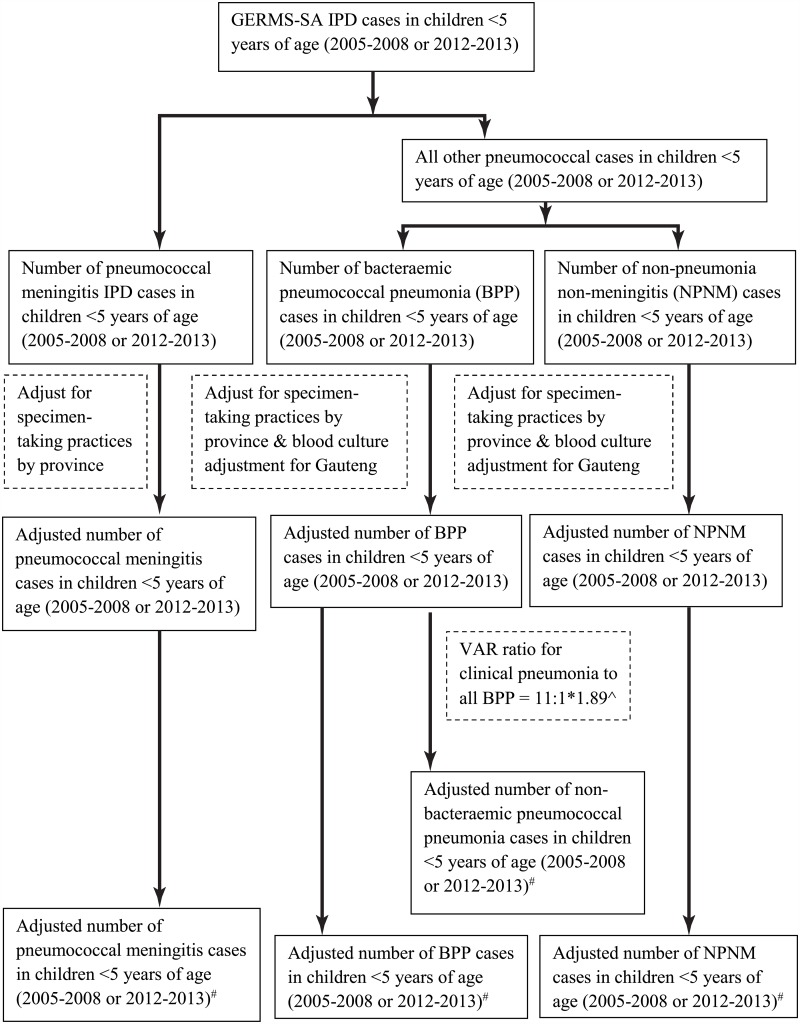
Flow diagram of the steps used to estimate the burden of invasive and non-invasive pneumococcal cases in children <5 years of age in South Africa in 2005–2008 and 2012–2013. Detailed figures are included in S1 Text. ^#^For all syndromes total case numbers as well as numbers stratified by HIV status were determined. ^Additional adjustment (1.89) for difference in VE estimated with use of urinary antigen.

We developed a conceptual model to estimate the national burden of pneumococcal cases, and deaths as well as associated rates, in children aged 0–59 months in South Africa for the baseline pre-vaccine era (an average estimate for the 2005–2008 period which had a stable disease incidence [11]) and a two-year period in the post-vaccine era (2012–2013) (Fig 1; S1 and S2 Figs). We used the observed cases of IPD hospitalisations from the GERMS-SA surveillance programme at both time points, stratified by disease syndrome (meningitis, pneumonia and non-pneumonia non-meningitis) and by age (<1 and 1–4 years of age) as the base rate and adjusted for incomplete specimen collection based on Corporate Data Warehouse (CDW) data by province among hospitalised children. Gauteng province was used as the reference province for this adjustment because of its systematic testing practices. We stratified all estimates by HIV status which has previously been documented to affect burden of disease [11]. Model parameters are given in Table 1 and calculations are explained further below as well as shown in Fig 1 (and S1 and S2 Figs). All reported case numbers were rounded to the closest 100, except when counts were less than 100, to reflect that case numbers were estimates and not exact numbers. Incidence rates were calculated from the estimated cases using population denominators from Statistics South Africa [20]; incidence rates were reported per 100,000 population without rounding off case numbers.

**Table 1:** Parameters used in base case model to estimate total number of cases, incidence and mortality rates for severe pneumococcal disease among children aged <5 years in South Africa.

Parameter	Value used in base case model	Source of data
Number of cases of invasive pneumococcal disease for 3 clinical syndromes (meningitis, BPP, NPNM)	IPD cases detected from enhanced and non-enhanced surveillance sites	GERMS-SA surveillance programme
Adjustment factor for systematic blood culturing from South African clinical trial: for Gauteng Province only	Ratio of BPP incidence (per 100,000 population) from Soweto clinical trial (1998–1999) over the average BPP incidence (per 100,000 population) from GERMS-SA surveillance in the same age group from 2005–2008 (≤2 years of age) = 22 overall, 13 in HIV-uninfected and 23 in HIV-infected children.	Madhi 2005: VE clinical trial conducted in Soweto located in the Gauteng Province
Adjustment for specimen-taking practices in provinces other than Gauteng Province	Provincial incidence rates were adjusted by the relative rate of blood cultures or CSF specimens collected in each province relative to Gauteng Province (baseline = 1). Rate ratios differed by province and specimen type. CSF specimen province specific rates: in 2005–2008 GA = 1.00, WC = 1.27, KZN = 2.00, NC = 1.86, EC = 2.81, NWP = 2.48, MP = 2.53, FS = 2.04, LP = 5.60 and in 2012–2013 GA = 1.00, WC = 0.23, KZN = 2.30, NC = 1.84, EC = 0.85, NWP = 1.66, MP = 2.71, FS = 0.39, LP = 5.70. Blood culture province specific rates: in 2005–2008 GA = 1.00, WC = 1.26, KZN = 6.00, NC = 4.32, EC = 4.58, NWP = 10.51, MP = 18.06, FS = 2.43, LP = 80.13 and in 2012–2013 GA = 1.00, WC = 0.35, KZN = 35.35, NC = 11.82, EC = 1.61, NWP = 15.08, MP = 40.06, FS = 10.71, LP = 82.37.	NHLS Corporate Data Warehouse (CDW): Collates data on CSF and blood specimens taken nationally & submitted to NHLS laboratories
Provincial incidences rates were calculated using provincial specific cases and provincial denominators $Rate\;ratio\;in\;other\;provinces\;=\;\frac{Rate\;in\;Gauteng\;Province}{Rate\;in\;other\;provinces}$		
Number of cases of NBP (NBP = BPP*11)	Ratio of PCV9 clinical pneumonia VAR (410 cases/100,000) to all BPP VAR (37 cases/100,000) = 11:1 –used to calculate number of NBP cases by multiplying ratio by BPP cases	Madhi 2005
HIV prevalence among IPD cases; used to calculate proportion of HIV-infected and—uninfected cases.	Number of HIV-infected and—uninfected cases calculated by syndrome and year	GERMS-SA surveillance programme—HIV data available for enhanced sites
Case fatality ratio CFR for bacteraemic syndromes	CFR for pneumococcal bacteraemic (meningitis, BPP, NMNP) syndromes = unadjusted pneumococcal deaths from enhanced sites/unadjusted pneumococcal cases from enhanced sites; CFR determined by age, HIV status and syndrome.	GERMS-SA surveillance data
CFR for non-bacteraemic pneumonia (2005–2008 period)	CFR for clinical LRTI in HIV-uninfected children = 1.9% (40/2139) and in HIV-infected children = 31% (281/899).	Madhi 2005
CFR for non-bacteraemic pneumonia (2012–2013 period)	CFR for presumed bacterial pneumonia in HIV-uninfected cases = 1% (13/1326) overall for cases with hospital controls (range 0.2% to 6.3% in different sites) and <1% (2/889) for cases with community controls.	Madhi 2015
Adjusted number of deaths	Adjusted number of pneumococcal deaths = CFR*Adjusted pneumococcal case numbers	GERMS-SA surveillance data
Incidence and death rates using mid-year population denominators	Incidence rates = Adjusted case numbers/population denominator Death rates = Adjusted death numbers/population denominator	Statistics South Africa data
HIV-specific denominators for incidence and death rates	Incidence and death rates by HIV status	Thembisa model (Johnson 2016)

BPP = Bacteraemic pneumococcal pneumonia; NPNM = Non-pneumonia non-meningitis; IPD = invasive pneumococcal disease; PCV9 = 9-valent pneumococcal conjugate vaccine; VAR = Vaccine-attributable reduction; VE = vaccine efficacy; CSF = cerebrospinal fluid; NHLS = National Health Laboratory Service; PCV13 = 13-valent PCV; CFR = case fatality ratio; NBP = Non-bacteraemic pneumococcal pneumonia

Provinces: GA = Gauteng, WC = Western Cape, KZN = KwaZulu-Natal, NC = Northern Cape, EC = Eastern Cape, NW = North West Province, MP = Mpumalanga, FS = Free State, LP = Limpopo Province

#### Estimated number of cases and incidence rates by clinical syndrome

IPD case numbers were estimated from national laboratory-based surveillance (described below) and divided into fractions attributable to meningitis, bacteraemic pneumonia and non-pneumonia non-meningitis cases on the basis of GERMS-SA clinical data. IPD case numbers were adjusted for incomplete specimen-taking practices. A direct adjustment was not made for sensitivity of blood culture among truly bacteraemic cases and CSF culture among those with pneumococcal meningitis. We assumed that the Gauteng Province had the most complete blood culturing practices and multiplied up the case numbers in the other provinces to calculate what the case numbers would have been if they had had the same complete blood culturing practices as that in Gauteng. The relevant provincial rate ratio was calculated by dividing the blood or CSF culture rate in the Gauteng Province by the rate in each individual province. To estimate the number of cases of non-bacteraemic pneumococcal pneumonia, we extrapolated data from PCV probe studies in South Africa [16] by using the PCV9 vaccine attributable reduction (VAR) ratio of clinical pneumonia to bacteraemic pneumococcal pneumonia (11:1), i.e. 11 clinical cases to 1 bacteraemic case (see S1 Text for detailed methods).

#### Estimated number of deaths and mortality rates

Pneumococcal death estimates for each syndrome were calculated by multiplying case estimates (as calculated above) by observed syndrome-specific case fatality ratios (CFRs) (from GERMS-SA enhanced sites) for IPD cases by age group and HIV status. For non-bacteraemic pneumonia parameters were taken from two South African studies. For the 2005–2008 model period we used data from a clinical trial conducted from 1998–2001. The CFR for clinical LRTI in HIV-uninfected children was 1.9% (40/2139) and in HIV-infected children was 31.3% (281/899) [16]. A later case-control study (2010–2012) for presumed bacterial pneumonia in HIV-uninfected cases showed a CFR of 1% (13/1326) overall for cases with hospital controls (range 0.2% to 6.3% in different sites) and <1% (2/889) for cases with community controls [22]. These CFRs were used for the 2012–2013 model period.

### Statistical analysis

We calculated the percent reduction in incidence and death rates between the two periods (2005–2008 and 2012–2013) using the following formulas:

$$\%\;reduction\;in\;incidence\;rates\;\left(IR\right)\;=\;\frac{{\bar{IR}}_{2005-2008}\;-\;{\bar{IR}}_{2012-2013}}{{\bar{IR}}_{2005-2008}}$$

$$\%\;reduction\;in\;mortality\;rates\;\left(MR\right)\;=\;\frac{{\bar{MR}}_{2005-2008}\;-\;{\bar{MR}}_{2012-2013}}{{\bar{MR}}_{2005-2008}}$$

Bootstrapping to create confidence intervals was used for all endpoints, to account for variability and uncertainty in detection rates, incidence rates and case-fatality rates from the surveillance data. For each model input/adjustment factors used in the calculations we obtained 1000 bootstrapped datasets and associated estimate providing and estimated variability of each model input/adjustment factor. This was done for either directly available data or those obtained from the published literature. The model calculation were then repeated using each of the 1000 bootstrapped datasets to propagate the level of variability associated with each model input/adjustment factor. We obtained the distribution for each parameter used in the model through resampling of the estimated parameter using the binomial distribution for proportions and the Poisson distribution for count data. We used reported measures for each of our estimates and defined the variance from 1000 resampled datasets obtained as described above. The lower and upper limits of the 95% CI were the 2.5th and 97.5th percentile of the 1000 model values obtained from the 1000 bootstrapped datasets.

### Human subjects review

Ethics approval was obtained for GERMS-SA surveillance (M081117) from the Human Research Ethics Committee (Medical), University of the Witwatersrand, Johannesburg, South Africa and other local hospital or provincial ethics committees, as required. Clearance for the surveillance programme was also obtained from the U.S. Centers for Disease Control and Prevention (IRB 00001223). The national surveillance programme included routine submission of laboratory isolates to NICD with basic demographic data; this did not require patient consent as it was part of the NICD’s national public health surveillance responsibility. At enhanced sites, additional data from patient interviews, was collected from participants who provide written informed consent. For children <18 years of age, written informed consent was obtained from parents or legal guardians. All patient identifiers were removed prior to data analysis.

### Sensitivity analysis

A one-way sensitivity analysis was performed by changing one variable at a time to see the effect on the total number of cases and deaths (parameters in Table 2). A Tornado diagram was fitted around the base case estimates for cases (S3 Fig) and deaths (S4 Fig) to evaluate the sensitivity of the model to changes in the assumed values of key parameters (See S1 Text).

**Table 2:** Parameters for sensitivity analysis for severe pneumococcal disease among children aged <5 years in South Africa.

Parameter	Value used in sensitivity model	Source of data
Number of cases of invasive pneumococcal disease for 3 clinical syndromes (meningitis, BPP, NPNM)	As for base calculations	GERMS-SA surveillance programme
Adjustment factor for systematic blood culturing from South African clinical trial: for Gauteng Province only	Ratio of IPD incidence from Soweto clinical trial (1998–1999) to the average IPD incidence from GERMS-SA surveillance in same age group from 2005–2008 (≤2 years of age) = 8.	Klugman 2003: VE clinical trial conducted in Soweto located in the Gauteng Province
Adjustment for specimen-taking practices in provinces other than Gauteng Province	As for base calculations	NHLS CDW
Number of cases of NBP (NBP = BPP*11*1.89)	PCV9 Clinical pneumonia VAR = 410 cases/100,000 and all BPP VAR = 37 cases/100,000 = ratio 11:1	Madhi 2005
	Additional adjustment for vaccine probe underestimate—VE against VT non-bacteraemic pneumonia is closer to 45% than 85% = 1.89	Bonten 2014
Number of cases of NBP (NBP = BPP*4*1.89)	PCV9 WHO CXR confirmed VAR = 155 cases/100,000 and all BPP VAR = 37 cases/100,000 = ratio 4:1 with adjustment factor (1.89) = ratio 7.6:1	Madhi 2005
Case fatality ratio of BPP to NBP	Adjusted risk ratio for death of end-point pneumonia (1.98) to the adjusted risk ratio for ‘other infiltrates/ abnormalities’ pneumonia (0.66) = 3:1	Enwere 2007
	CFR for non-bacteraemic pneumococcal pneumonia based on published data on difference in CFR (28.2%) between hospitalised all-cause bacteraemic cases and non-bacteraemic acute medical cases (5.7%) in Kenya = 5:1.	Berkley 2005
Adjusted number of deaths	Adjusted number of pneumococcal deaths = CFR*Adjusted pneumococcal case numbers—used different CFR	GERMS-SA surveillance programme
Deaths in the community	(Deaths by syndrome outside hospital/Deaths by syndrome in-hospital)*(Deaths from GERMS-SA enhanced sites) by age, syndrome and year	Vital statistics data from Statistics South Africa
Mid-year population denominators for incidence and death rates	As for base case model	Statistics South Africa data
HIV-specific denominators for incidence and death rates	As for base case model	Thembisa model (Johnson 2016)

BPP = Bacteraemic pneumococcal pneumonia; NPNM = Non-pneumonia non-meningitis; IPD = invasive pneumococcal disease; PCV9 = 9-valent pneumococcal conjugate vaccine; VAR = Vaccine-attributable reduction; VE = vaccine efficacy; CSF = cerebrospinal fluid; NHLS = National Health Laboratory Service; PCV13 = 13-valent PCV; CFR = case fatality ratio; NBP = Non-bacteraemic pneumococcal pneumonia

Provinces: GA = Gauteng, WC = Western Cape, KZN = KwaZulu-Natal, NC = Northern Cape, EC = Eastern Cape, NW = North West Province, MP = Mpumalanga, FS = Free State, LP = Limpopo Province

## Results

### Burden of invasive pneumococcal disease in the pre-vaccine era

In the pre-vaccine era (2005–2008), an estimated national average of 107,600 (83,000–140,000) annual cases of hospitalised pneumococcal disease, corresponding to an incidence of 2,074 (1,603–2,730) per 100,000 person-years (py), occurred in children aged 0–59 months in South Africa (Tables 3 and 4). An average of 1,100 (1,000–1,200) cases of pneumococcal meningitis (21 per 100,000 py); 8,600 (6700–11,200) bacteraemic pneumococcal pneumonia cases (163 per 100,000 py), 93,000 (71,700–123,000) non-bacteraemic pneumococcal pneumonia cases (1,797 per 100,000 py) and 4,900 (3,600–6,100) non-pneumonia non-meningitis invasive pneumococcal disease cases (93 per 100,000 py) were estimated to occur annually in children aged 0–59 months. Based on model inputs, the overall incidence for hospitalised pneumococcal disease was higher amongst infants <1 year of age (4,952 per 100,000 py) than children aged 1–4 years (1,343 per 100,000 py), a relative risk of 3.69 (95% CI 3.64–3.71); incidence was also higher among HIV-infected children (29,159 per 100,000 py) than among HIV-uninfected children aged 0–59 months (891 per 100,000 py), a relative risk of 33 (95% CI 30–34). Similar trends were observed in all syndromes (Table 4).

**Table 3:** Number of pneumococcal cases, by syndrome, in South Africa, by age and HIV status, 2005–2008 and 2012–2013.

	Total			HIV-infected			HIV-uninfected		
Syndrome/age group	Mean case numbers 2005–2008^#^ (95% CI)	Mean case numbers 2012–2013^#^ (95% CI)	Reduction in cases*	Mean case numbers 2005–2008^#^ (95% CI)	Mean case numbers 2012–2013^#^ (95% CI)	Reduction in cases*	Mean case numbers 2005–2008^#^ (95% CI)	Mean case numbers 2012–2013^#^ (95% CI)	Reduction in cases*
**Pneumococcal meningitis cases**									
**<1 year**	700 (680–740)	190 (180–240)	510 (500–540)	370 (320–390)	40 (20–60)	330 (300–360)	350 (330–390)	150 (130–180)	200 (170–210)
**1–4 years**	370 (350–390)	60 (50–70)	310 (300–320)	230 (210–250)	30 (20–40)	200 (190–220)	140 (120–160)	30 (10–40)	110 (110–130)
**<5 years**	1100 (1000–1200)	250 (230–280)	850 (770–920)	600 (540–630)	70 (50–80)	530 (490–550)	490 (460–540)	180 (160–220)	310 (280–320)
**Bacteraemic pneumococcal pneumonia cases**									
**<1 year**	4100 (3000–5000)	1700 (1100–2200)	2400 (1900–2800)	2200 (1600–2700)	400 (150–550)	1800 (1450–2150)	1900 (1500–2600)	1300 (800–1700)	600 (600–900)
**1–4 years**	4500 (3500–5900)	1800 (1100–2100)	2700 (2400–3800)	2800 (2100–3600)	800 (500–1000)	2000 (1600–2600)	1700 (1300–2300)	960 (600–1200)	740 (700–1100)
**<5 years**	8600 (6700–11200)	3500 (2000–4000)	5100 (4700–7200)	5000 (3800–6500)	1200 (700–1500)	3800 (3100–5000)	3600 (2800–4800)	2300 (1500–3000)	1300 (1100–2000)
**Non-bacteraemic pneumococcal pneumonia cases**									
**<1 year**	44000 (34000–58000)	18000 (12000–23000)	26000 (42600–66400)	24000 (16000–29800)	4100 (1800–6000)	19900 (14200–23800)	20000 (16800–29700)	14000 (9500–18300)	6000 (12700–17700)
**1–4 years**	49000 (37300–64400)	19000 (12500–22400)	30000 (50200–72100)	31000 (23000–41000)	8600 (4900–10500)	22400 (18100–30500)	18000 (13900–25100)	10600 (6900–13000)	7400 (14800–23000)
**<5 years**	93000 (71700–123000)	37000 (25000–45000)	56000 (93000–147000)	55000 (40400–69400)	12700 (6800–14800)	42300 (33600–54600)	38000 (30800–53500)	24600 (17400–32000)	13400 (27600–39500)
**Non-pneumonia non-meningitis invasive pneumococcal cases**									
**<1 year**	2800 (2100–3600)	500 (400–800)	2300 (1700–2800)	1500 (1000–1800)	100 (50–200)	1400 (1000–1600)	1200 (1000–1800)	400 (300–650)	800 (700–1100)
**1–4 years**	2100 (1500–2600)	500 (400–800)	1600 (1100–1800)	1300 (900–1700)	200 (150–350)	1100 (750–1300)	800 (600–1000)	300 (200–450)	500 (400–600)
**<5 years**	4900 (3500–6200)	1000 (800–1500)	3900 (2800–4600)	2900 (2000–3500)	350 (200–500)	2500 (1800–3000)	2000 (1500–2700)	600 (500–900)	1400 (1100–1700)
**Total pneumococcal cases**									
**<1 year**	51600 (40000–68000)	20400 (15000–26000)	31200 (46400–71700)	28100 (19500–34400)	4600 (2700–7200)	23400 (17000–27900)	23500 (19800–34400)	15900 (12500–23000)	7600 (7000–11400)
**1–4 years**	56000 (43000–73000)	21400 (14000–25000)	34600 (52900–82600)	35400 (26200–46400)	9600 (5800–11900)	25700 (20600–34600)	20600 (15900–28400)	11900 (7900–15000)	8700 (8000–13400)
**<5 years**	107600 (83000–140000)	41800 (28000–50000)	65800 (99000–156000)	63500 (46600–79800)	14300 (8500–19000)	49100 (39000–63200)	44100 (35400–62800)	27700 (20400–38000)	16400 (15000–24800)

CI = confidence interval; BPP = Bacteraemic pneumococcal pneumonia; NBP = Non-bacteraemic pneumococcal pneumonia; NPNM = Non-pneumonia non-meningitis

^**#**^Average number of cases per year;

*Reduction in cases = difference in case numbers between 2005–2008 and 2012–2013

**Table 4:** Pneumococcal incidence rates in South Africa, by syndrome, age and HIV status, 2005–2008 and 2012–2013.

	Total			HIV-infected (HI)			HIV-uninfected (HU)			IRR^	
Syndrome and age group	Incidence rate*2005–2008 (95% CI)	Incidence rate* 2012–2013 (95% CI)	% reduction	Incidence rate* 2005–2008 (95% CI)	Incidence rate* 2012–2013 (95% CI)	% reduction	Incidence rate* 2005–2008 (95% CI)	Incidence rate* 2012–2013 (95% CI)	% reduction	IRR^ HI/HU 2005–2008 (95%CI)	IRR^ HI/HU 2012–2013 (95%CI)
**Pneumococcal meningitis rates**											
**<1 year**	68 (66–71)	18 (16–20)	72 (68–76)	1155 (965–1165)	450 (226–643)	60 (45–77)	35 (32–38)	14 (12–16)	60 (58–63)	33 (27–37)	32 (20–40)
**1–4 years**	9 (8–10)	1.5 (1.3–1.8)	88 (82–90)	129 (117–139)	30 (18–38)	77 (71–85)	3.5 (3–4)	0.6 (0.4–0.8)	83 (80–87)	37 (29–44)	50 (25–88)
**<5 years**	21 (20–22)	5 (4–6)	77 (74–81)	288 (252–289)	72 (54–97)	75 (66–79)	10 (9–11)	3 (2–4)	70 (64–77)	29 (25–32)	24 (14–26)
**Bacteraemic pneumococcal pneumonia rates**											
**<1 year**	386 (300–509)	152 (102–200)	62 (60–65)	6516 (4539–7987)	3854 (1588–6177)	46 (41–54)	182 (150–262)	119 (78–160)	35 (32–41)	36 (33–37)	32 (26–34)
**1–4 years**	107 (86–142)	42 (27–51)	69 (67–71)	1523 (1161–1988)	933 (547–1200)	44 (40–50)	41 (33–57)	23 (13–29)	56 (53–60)	37 (34–39)	41 (40–52)
**<5 years**	163 (129–218)	64 (42–80)	65 (64–67)	2298 (1689–2906)	1233 (755–1693)	51 (49–55)	70 (56–97)	43 (28–56)	44 (43–49)	33 (31–34)	29 (27–31)
**Non-bacteraemic pneumococcal pneumonia rates**											
**<1 year**	4242 (3274–5574)	1674 (1145–2078)	61 (62–64)	71675 (49143–88925)	42391 (18885–62429)	41 (30–62)	2007 (1664–2931)	1310 (877–1692)	35 (32–47)	36 (34–37)	32 (29–37)
**1–4 years**	1176 (906–1566)	458 (298–531)	61 (68–70)	16757 (12596–22257)	10260 (5923–12537)	39 (44–53)	453 (354–638)	258 (167–324)	43 (39–53)	37 (36–38)	40 (35–41)
**<5 years**	1797 (1388–2379)	708 (473–843)	61 (65–67)	25281 (18672–32129)	13568 (7250–15696)	46 (32–61)	771 (622–1081)	476 (334–616)	38 (35–47)	33 (32–34)	29 (28–30)
**Non-pneumonia non-meningitis invasive pneumococcal rates**											
**<1 year**	256 (196–341)	49 (36–73)	81 (79–82)	4333 (2987–5467)	1245 (610–2065)	71 (65–77)	121 (100–177)	38 (27–59)	68 (62–69)	36 (32–38)	33 (23–35)
**1–4 years**	51 (35–64)	13 (9–18)	75 (72–76)	733 (489–905)	281 (180–420)	62 (54–64)	20 (14–26)	7 (5–11)	64 (55–68)	37 (35–40)	40 (36–48)
**<5 years**	93 (68–120)	20 (15–28)	78 (74–79)	1292 (900–1606)	381 (254–548)	71 (66–73)	41 (31–55)	14 (10–20)	67 (63–68)	32 (30–34)	27 (25–28)
**Total pneumococcal rates**											
**<1 year**	4952 (3838–6483)	1893 (1306–2353)	62 (60–66)	83679 (58197–102645)	47937 (28012–74881)	43 (27–52)	2343 (1956–3403)	1482 (1154–2116)	38 (37–39)	36 (34–37)	32 (24–35)
**1–4 years**	1343 (1035–1782)	513 (338–599)	62 (60–70)	19142 (14378–25398)	11506 (6853–14018)	40 (35–52)	518 (404–721)	289 (191–366)	44 (39–53)	37 (36–38)	40 (36–48)
**<5 years**	2074 (1603–2730)	797 (537–951)	62 (61–65)	29159 (21578–36924)	15257 (8567–17469)	48 (43–60)	891 (717–1237)	537(377–687)	47 (46–48)	33 (30–34)	28 (23–30)

*Per 100,000 population; CI = confidence interval;

^IRR = incidence rate ratio; IPD = invasive pneumococcal disease; BPP = Bacteraemic pneumococcal pneumonia; NBP = Non-bacteraemic pneumococcal pneumonia; NPNM = Non-pneumonia non-meningitis

### Pneumococcal related deaths and mortality rates in the pre-vaccine era

In the pre-vaccine period, an average of 5,000 (3,000–6,000) annual pneumococcal-related deaths, translating into a mortality rate of 97 per 100,000 py, was estimated to have occurred in children aged 0–59 months (Tables 5 and 6). An average of 370 (300–400) pneumococcal meningitis deaths (7 per 100,000 py), 1,300 (900–2,000) bacteraemic pneumococcal pneumonia deaths (26 per 100,000 py), 2,300 (1,500–2,500) non-bacteraemic pneumococcal pneumonia deaths (45 per 100,000 py) and 1,000 (200–2,000) non-pneumonia non-meningitis IPD deaths (19 per 100,000 py) were estimated per year. The overall pneumococcal mortality rate, based on CFRs, was higher amongst infants (303 per 100,000 py) than children aged 1–4 years (45 per 100,000 py), a relative risk of 6.73 (95% CI 6.23–7.03), and also higher amongst HIV-infected children (1,339 per 100,000 py) than amongst HIV-uninfected children aged 0–59 months (43 per 100,000 py), a relative risk of 31 (95% CI 29–32) (Table 6).

**Table 5:** Number of pneumococcal deaths in South Africa, by syndrome, age and HIV status, 2005–2008 and 2012–2013.

	Total			HIV-infected			HIV-uninfected		
Syndrome/age group	Mean number of deaths 2005–2008 (95% confidence interval (CI)	Mean number of deaths 2012–2013 (95% CI)	Reduction in deaths (2005/8-2012/3)	Mean number of deaths 2005–2008 (95% CI)	Mean number of deaths 2012–2013 (95% CI)	Reduction in deaths (2005/8-2012/3)	Mean number of deaths 2005–2008 (95% CI)	Mean number of deaths 2012–2013 (95% CI)	Reduction in deaths (2005/8-2012/3)
**Pneumococcal meningitis deaths**									
**<1 year**	300 (200–400)	60 (30–90)	240 (170–310)	160 (130–270)	15 (4–25)	145 (86–165)	110 (90–180)	50 (20–90)	60 (70–90)
**1–4 years**	90 (50–130)	20 (10–40)	70 (40–90)	60 (10–110)	10 (4–20)	50 (10–90)	30 (10–60)	10 (5–20)	20 (15–40)
**<5 years**	370 (300–400)	80 (50–100)	290 (250–300)	200 (100–300)	20 (10–40)	180 (90–260)	150 (100–200)	60 (40–100)	90 (60–150)
**Bacteraemic pneumococcal pneumonia deaths**									
**<1 year**	900 (300–1600)	400 (70–900)	500 (200–700)	500 (200–800)	90 (20–220)	410 (80–580)	400 (200–800)	300 (100–700)	100 (80–120)
**1–4 years**	400 (150–900)	200 (0–700)	200 (150–250)	300 (0–800)	100 (0–300)	200 (0–500)	200 (0–500)	100 (0–400)	100 (0–150)
**<5 years**	1300 (900–2000)	600 (250–1200)	700 (650–800)	800 (300–1400)	200 (40–400)	600 (260–1000)	600 (200–1000)	400 (90–800)	200 (110–290)
**Non-bacteraemic pneumococcal pneumonia deaths**									
**<1 year**	1300 (1000–1700)	550 (400–700)	750 (600–1000)	700 (500–900)	100 (50–200)	600 (450–700)	600 (500–900)	400 (300–500)	200 (150–400)
**1–4 years**	1000 (800–1300)	400 (250–450)	600 (550–850)	600 (500–800)	150 (100–200)	450 (400–600)	400 (300–500)	200 (150–250)	200 (150–250)
**<5 years**	2300 (1500–2500)	950 (500–1000)	1350 (1000–1500)	1300 (800–1400)	300 (100–350)	1000 (700–1050)	1000 (600–1200)	600 (350–650)	400 (250–550)
**Non-pneumonia non-meningitis invasive pneumococcal deaths**									
**<1 year**	700 (150–1500)	200 (0–400)	500 (150–1100)	400 (70–700)	40 (0–100)	360 (70–600)	300 (70–700)	100 (0–300)	200 (70–400)
**1–4 years**	300 (0–950)	50 (0–350)	250 (0–600)	200 (0–600)	20 (0–150)	180 (0–450)	100 (0–400)	30 (0–200)	70 (0–200)
**<5 years**	1000 (200–2000)	300 (0–600)	700 (200–1400)	600 (100–1000)	60 (0–200)	540 (100–800)	400 (100–800)	200 (0–450)	200 (100–350)
**Total pneumococcal deaths**									
**<1 year**	3000 (2000–4000)	1200 (700–1700)	1800 (1300–2300)	1700 (1000–2300)	300 (200–700)	1400 (800–1600)	1400 (1000–2200)	900 (700–1500)	500 (300–700)
**1–4 years**	2000 (1200–2800)	700 (300–1100)	1300 (900–1700)	1200 (700–2000)	300 (100–500)	900 (600–1500)	800 (400–1000)	400 (200–700)	400 (200–400)
**<5 years**	5000 (3000–6000)	1900 (1000–2500)	3100 (2000–3500)	3000 (1800–3600)	600 (300–800)	2300 (1500–2800)	2200 (1400–2800)	1300 (700–1800)	900 (700–1000)

CI = confidence interval

**Table 6:** Pneumococcal mortality rate in South Africa, by syndrome, age and HIV status, 2005–2008 and 2012–2013.

	Total			HIV-infected (HI)			HIV-uninfected (HU)			Incidence rate ratio	
Syndrome/age group	MR* 2005–2008 (95% CI)	MR* 2012–2013 (95% CI)	% reduction	MR* 2005–2008 (95% CI)	MR* 2012–2013 (95% CI)	% reduction	MR* 2005–2008 (95% CI)	MR* 2012–2013 (95% CI)	% reduction	MRR HI/HU 2005–2008 (95% CI)	MRR HI/HU 2012–2013 (95% CI)
**Pneumococcal meningitis rates**											
**<1 year**	26 (21–31)	6 (2–10)	79 (70–83)	490 (261–548)	140 (36–291)	71 (42–83)	11 (8–19)	4 (2–8)	60 (46–73)	37 (28–46)	28 (15–57)
**1–4 years**	2 (1–3)	0.6(0.2–1)	77 (60–85)	32 (7–59)	12 (5–26)	64 (15–79)	1 (0.2–2)	0.3 (0.1–0.5)	66 (29–82)	32 (24–59)	47 (18–109)
**<5 years**	7 (6–8)	2 (1–3)	78 (70–81)	103 (62–126)	25 (13–45)	76 (55–82)	3 (2–5)	1 (0.7–2)	62 (47–71)	32 (24–36)	27 (13–35)
**Bacteraemic pneumococcal pneumonia rates**											
**<1 year**	86 (31–155)	38 (19–50)	55 (49–59)	1457 (480–2454)	973 (221–2248)	33 (18–49)	41 (15–78)	30 (7–66)	26 (10–32)	36 (31–40)	29 (23–38)
**1–4 years**	11 (4–21)	5 (0–16)	50 (54–68)	153 (0–424)	121 (0–448)	21 (12–47)	4 (0–12)	3 (0–10)	27 (21–59)	39 (30–45)	54 (35–64)
**<5 years**	26 (17–40)	12 (2–24)	53 (53–61)	355 (137–655)	209 (47–486)	41 (36–55)	12 (4–22)	9 (2–17)	25 (21–39)	30 (28–34)	25 (20–28)
**Non-bacteraemic pneumococcal pneumonia rates**											
**<1 year**	127 (98–167)	50 (38–67)	61 (59–67)	2150 (1474–2667)	1272 (566–1872)	41 (30–60)	60 (50–88)	39 (26–51)	35 (32–48)	36 (30–38)	33 (22–36)
**1–4 years**	24 (18–31)	9 (5–15)	61 (50–75)	335 (251–445)	205 (118–251)	39 (37–53)	9 (7–13)	5 (3–6)	44 (42–57)	37 (34–41)	41 (39–42)
**<5 years**	45 (28–48)	18 (10–26)	60 (50–64)	617 (373–642)	315 (145–333)	49 (47–61)	20 (12–22)	12 (6–16)	37 (28–49)	31 (29–32)	23 (21–24)
**Non-pneumonia non-meningitis invasive pneumococcal rates**											
**<1 year**	63 (14–141)	16 (5–42)	74 (62–72)	1069 (223–2172)	415 (0–1000)	61 (38–68)	30 (7–68)	13 (0–30)	57 (55–75)	35 (31–42)	29 (21–41)
**1–4 years**	8 (0–23)	1 (0–8)	85 (80–89)	115 (0–320)	26 (0–184)	77 (60–83)	3 (0–9)	1 (0–5)	79 (69–87)	39 (30–46)	31 (26–85)
**<5 years**	19 (4–35)	4 (0–12)	78 (70–77)	263 (50–472)	66 (0–232)	75 (62–78)	9 (2–16)	3 (0–9)	63 (47–68)	30 (27–35)	20 (15–26)
**Total pneumococcal rates**											
**<1 year**	303 (204–424)	111 (77–216)	64 (51–62)	5166 (3177–6733)	2800 (1565–4511)	46 (33–51)	142 (105–221)	87 (62–174)	39 (22–31)	36 (30–38)	32 (25–33)
**1–4 years**	45 (29–68)	16 (10–34)	64 (50–66)	636 (364–1048)	364 (132–614)	43 (41–64)	17 (10–30)	9 (4–17)	47 (43–56)	37 (34–40)	40 (33–53)
**<5 years**	97 (63–117)	36 (19–58)	63 (51–69)	1339 (829–1651)	615 (274–797)	54 (51–67)	43 (28–56)	25 (13–33)	41 (37–53)	31 (29–32)	25 (21–26)

*Mortality rate (MR) per 100,000; MRR = Mortality rate ratio; CI = confidence interval

### Impact of the pneumococcal conjugate vaccine and other interventions on the burden of disease in 2012–2013

Based on inputted model parameters we estimated that in 2012–2013 there was an average of 41,800 (28,000–50,000) pneumococcal cases in children aged 0–59 months, 65,800 fewer cases than would have been expected based on the incidence of disease observed in 2005–2008 among this age group (Table 3). The overall national annual incidence of pneumococcal disease in 2012–2013 was estimated as 797 per 100,000 py in children 0–59 months of age, a total rate difference of 1,277 per 100,000 (62% reduction) compared with the pre-PCV period (Table 4). The annual incidence of pneumococcal meningitis in 2012–2013 was 5 per 100,000 py in children aged 0–59 months (rate difference of 16 per 100,000, 77% reduction), 64 per 100,000 py for bacteraemic pneumococcal pneumonia (rate difference of 99 per 100,000, 65% reduction) and 708 per 100,000 py for non-bacteraemic pneumococcal pneumonia (rate difference of 1,089 per 100,000, 61% reduction). For all syndromes incidence was highest amongst infants and HIV-infected children as is expected based on the rates observed in the IPD GERMS data.

### Pneumococcal related deaths and mortality rates in 2012–2013

In 2012–2013 we estimated 1,900 (1,000–2,500) annual pneumococcal deaths in children aged 0–59 months, 3,100 fewer than would have been expected based on modelled pneumococcal deaths in 2005–2008 (Table 5). The overall South African annual mortality rate for pneumococcal disease in 2012–2013 was estimated at 36 per 100,000 py in children aged 0–59 months, a rate difference of 61 per 100,000 py (63% reduction) compared with the pre-PCV years (Table 6). The mortality rate was 6.94 (6.35–7.70) times greater in infants (111 per 100,000 py) than in children aged 1–4 years (16 per 100,000 py). The average pneumococcal meningitis mortality rate in 2012–2013 was 2 per 100,000 py in children aged 0–59 months, a rate difference of 5 per 100,000 (78% reduction) compared with the 2005–2008 rate; 12 per 100,000 py for bacteraemic pneumococcal pneumonia (rate difference of 14 per 100,000, 53% reduction) and 18 per 100,000 py for non-bacteraemic pneumococcal pneumonia (rate difference of 27 per 100,000, 60% reduction).

### Sensitivity analysis

The total numbers of pneumococcal cases and deaths estimated by the model changed depending on the values of key parameters used in the model (Table 2); variations resulted in both higher and lower estimates of cases and deaths, showing the importance of using the best estimates (S2 and S3 Tables; Tornado diagrams, S3 and S4 Figs). For the pre-vaccine estimates, the inclusion of death estimates in the community did not change the number of cases (109,500) and deaths (3700) significantly from our base model (107,600 cases and 5000 deaths), while variations in the CFR resulted in an increase in the estimated number of pneumococcal deaths and separate HIV estimates doubled the death rates likely fully accounting for HIV-infected rates. In the sensitivity analysis when we changed other parameters, including the VAR NBP/BPP ratio of 7.6:1 and the adjustment factor for systematic blood culturing the case numbers were reduced by 25% and 9% respectively. When the same parameters were altered in estimating pneumococcal deaths, numbers were reduced by 8% and 32%. Adding an additional adjustment to account for the lack of sensitivity of determining the VE in pneumonia increased the case estimates by 83% and the death estimates by 23% as a higher proportion of pneumonia was assumed to have been caused by the pneumococcus.

## Discussion

Our South African pneumococcal disease burden model has estimated that in the pre-vaccine era (2005–2008) an average of 107,600 (83,000–140,000) cases of severe pneumococcal disease were experienced per year in children aged 0–59 months. In 2012–2013, 41,800 cases were estimated, a 1,277 per 100,000 py rate difference. This 62% reduction in all serotype IPD compared with a non-PCV period was likely due to PCV introduction as well as improvements in HIV care and prevention. Despite the elevated relative risk of IPD in HIV-infected children, the low HIV prevalence in children may mean that HIV prevention has a limited effect on the reduction of pneumococcal disease at a population level. Other studies in the PCV13 era, which compared reductions with the PCV7 period, showed a 64% (95% CI 59–68%) reduction in all IPD in the USA in children aged <5 years [23] and in the Gambia a 55% (95% CI 30–71%) reduction in children aged 2–23 months and 56% (95% CI 25–75%) reduction in children aged 2–4 years [24]. In the UK in 2014/2015 in all age groups the overall incidence of IPD, compared to the pre-PCV7 period, declined by 47%, but an increase was noted in non-PCV13 serotypes in this period [25].

The model estimated 5,000 (3,000–6,000) annual pneumococcal deaths in children aged 0–59 months in the pre-vaccine era; this translated into a mortality rate of 97 per 100,000 py. In children aged 0–59 months in South Africa there was an average of 61,749 annual all cause deaths, 19,072 annual all respiratory deaths and 14,927 annual pneumonia and influenza deaths over the 2005–2008 period based on Statistics South Africa data [26]; the estimated pneumococcal deaths would have made up 8%, 26% and 33% of these deaths respectively. A meta-analysis, including studies from the US, South Africa, Gambia and the Philippines, which assessed PCV efficacy on pneumonia, concluded that approximately 21.2% of severe clinical pneumonia and 35.8% of chest radiograph (CXR) confirmed pneumonia was attributable to pneumococcal disease [7]; the clinical trial estimates included lower-valency vaccines and the Philippines study included a lower efficacy vaccine. In 2012–2013 we estimated that an average of 1,900 (1,000–2,500) annual pneumococcal-related deaths occurred in children aged 0–59 months, a mortality rate reduction of 61 per 100,000 py. In 2012–2013, in children <5 years there were an estimated 35,989 overall deaths, 8,720 all respiratory and 3,946 annual pneumonia and influenza deaths per annum in South Africa, based on Statistics South Africa data [26]; the estimated pneumococcal deaths would have contributed to 5%, 22% and 48% of these deaths respectively. A review by Izadnegahdar, et. al. in 2013 [27] proposed that even in the post-PCV13 period, *S*. *pneumoniae* pneumonia may still make up 44% of pneumonia deaths due to non-PCV13 serotypes. This estimate is higher than that reported in a Child Health Epidemiology Reference Group (CHERG) systematic review which estimated that in 2011 *S*. *pneumoniae* made up 32.7% of pneumonia deaths in the African region and globally [28].

An updated global burden model that includes a time series from 2000–2015 [1] with annual rates, calculated a total pneumococcal death rate of 203 (164–241) per 100,000 py, 19 (16–23) per 100,000 py for pneumococcal meningitis and 166 (133–198) per 100,000 py for pneumococcal pneumonia in children aged 1–59 months in 2008 for South Africa. When compared with our model from the pre-PCV era which included children 0–59 months, the point estimates for the global model were higher than all our rates; our overall rates, 97 (63–117) per 100,000 py; pneumonia rates, 71 (45–88) per 100,000 py, and calculated meningitis rates, 7 (6–8) per 100,000 py. In 2012–2013, our overall rates, 36 (19–58) per 100,000 py and pneumonia rates, 30 (12–50) per 100,000 py, were still slightly lower than the South African estimates from the global model, 70 (57–83) and 56 (45–66) per 100,000 py respectively. The two models differed in their conceptual approach, their approach to neonatal deaths, and the inclusion of different input parameters, including case fatality rates which likely accounts for the difference in death rate estimates. The global disease burden model is centred on a proportional mortality approach, taking as a given the all-pathogen deaths for meningitis and for pneumonia provided by the Maternal and Child Epidemiology Estimation (MCEE) Group. These deaths are apportioned out to pneumococcus using country specific empirical data for meningitis and PCV clinical trial data (for pneumonia). In contrast, the South Africa specific model used a ‘bottom-up’ approach using IPD cases from surveillance data as the anchor for the estimates, but it used the same PCV clinical trial data for pneumonia estimates. Lastly our model used HIV prevalence rates differently to other models; our model used HIV prevalence rates for children identified with pneumococcal disease from our surveillance programme, while other models usually apply community HIV prevalence rates.

Stratifying data by HIV status revealed a 47% reduction in the incidence of all serotype pneumococcal disease in HIV-uninfected children, and a 48% reduction in HIV-infected children aged 0–59 months based on data inputted into the model. Based on observations from IPD trends in South Africa, and assuming that these IPD trends reflect all pneumococcal trends, it is likely that all of the reductions in HIV-uninfected children and approximately half of the reductions in HIV-infected children may be attributable to the vaccine [6]. In HIV-infected children antiretroviral therapy and improvements in the prevention of mother-to-child transmission of HIV programme, contribute to reductions in all serotypes.

Since the IPD syndromic distribution observed in 2005–2008 and in 2012–2013 drives the case and death estimates for these two periods, any reductions by syndrome are inherently a result of the differences observed by syndrome in the IPD cases. As a result of those differences, the model output had reductions in all syndromes between the pre- and the post-PCV periods. Similarly reductions were greatest in infants and HIV-infected children, the latter driven by the relative risk inputted into the model. Similarly the overall pneumococcal mortality rate, which was driven by CFRs inputted into the model, was higher amongst infants.

This study is subject to a number of limitations. First, the model is anchored on the GERMS-SA IPD surveillance data which is primarily drawn from public sector laboratories, so may not be representative of all sectors in South Africa; however, 84% of the population access public health care in South Africa [13]. The surveillance programme aims to include all cases including those detected in private laboratories however not all private sector cases may be captured. Second, a number of assumptions were made to adjust for the lack of sensitivity of detecting cases. Although all the assumptions were based on published literature, it is possible that some of these assumptions were not accurate. We could also not find data on all possible model parameters, for example the relative risk of pneumococcal disease in HIV-infected children pre- and post-ART introduction. To simplify the model some estimates, for example VARs, were used for all patients even though in reality there may be some differences by age and HIV status. Some estimates were only available for the pre-vaccine period and were assumed to be relevant to the post-PCV period, which may have underestimated the reduction in disease in the post-PCV period. The variation in our estimates was shown in our sensitivity analysis which demonstrated the wide range of results possible by varying key parameters. This shows the importance of using best estimates for key values. Third, we only calculated the burden of severe pneumococcal disease and did not include pneumococcal disease that was cared for only in the outpatient setting. We were unable to include otitis media burden calculations in this model due to a lack of reliable African data. A US study showed that in children <5 years of age, acute otitis media made up 74% of pneumococcal cases [29], so this burden model is an underestimate of true pneumococcal burden. Fourth, as we used adjustment factors from a PCV-probe study in children [16] which based the diagnosis of pneumonia on clinical and CXR findings only, both of which have limitations in detection, we may have underestimated the burden of non-bacteraemic pneumonia in our calculations. Fifth, we assumed similar PCV impact across all age strata among children less than 5 years and did not account for direct or indirect vaccine effects separately; we assumed that by using actual reported cases this would account for different impact rates. Sixth, there was a reduction in all-cause pneumonia deaths in South Africa over the 2005 to 2008 period [26]; for the pre-PCV death rate calculations we assumed that rates were similar over this period and we may have therefore overestimated the change in pneumococcal death rates when we compared the average and not the end of the pre-vaccine period (2008) with 2012–2013. Seventh, we included neonates as part of the <1 year old age group and did not separate them out due to small case numbers and lack of specific adjustment parameters for this group only. Although the epidemiology of disease in neonates differs somewhat from older children, leaving this group out may have underestimated the total burden of disease. Eighth, rates of disease are not entirely independent of pneumococcal disease and it is possible that provinces with higher rates of culture also have higher rates of pneumococcal disease [30]. Lastly, we used bootstrapping to generate confidence intervals to try and account for some of the uncertainty around our estimates; however some external parameters derived from different settings and the full scope of variability may not be fully represented by our assumptions. We attempted to reduce this by including as many factors as possible in our bootstrap resampling and running 1,000 replications. It is however likely that the confidence intervals around case count and impact estimates are narrower than in reality.

GERMS-SA IPD surveillance data demonstrated a reduction in vaccine-type IPD of around 50% among adults aged 25 to 44 years by 2012 [6]. The indirect vaccination effects were similar in HIV-infected (40%) and HIV-uninfected (52%) adults. It would be useful to calculate similar burden estimates, as in this paper, for individuals ≥5 years of age.

In summary, pneumococcal disease represents a major public health burden in children aged 0–59 months in South Africa. Pneumococcal conjugate vaccination, in conjunction with other interventions, has resulted in a significant reduction in severe pneumococcal disease with approximately 130,000 cases and 5,000 deaths averted over a 5-year period. Although other interventions likely contribute to reductions in pneumococcal disease it is possible that PCV use was the major contributor to the reduction in invasive disease.

## Supporting information

S1 Text Supplementary material: Estimated severe pneumococcal disease cases and deaths before and after pneumococcal conjugate vaccine introduction in children younger than 5 years of age in South Africa. (DOCX)

S1 Table Population denominators from the Thembisa model for children <5 years of age in South Africa, 2005–2008 and 2012–2013. (DOCX)

S2 Table Sensitivity analysis for case numbers showing key variables altered in analysis, 2005–2008 and 2012–2013. (DOCX)

S3 Table Sensitivity analysis for numbers of deaths showing key variables altered in analysis, 2005–2008 and 2012–2013. (DOCX)

S1 Fig Initial step in estimating the burden of invasive and non-invasive pneumococcal cases in children aged <5 years in South Africa, 2005–2008 and 2012–2013. (EPS)

S2 Fig Second step in estimating the burden of invasive and non-invasive pneumococcal cases in children <5 years in South Africa, 2005–2008 and 2012–2013. (EPS)

S3 Fig Tornado sensitivity diagram representing change in pneumococcal case estimates in children <5 years of age in the pre-vaccine era, when values of key variables are modified. (EPS)

S4 Fig Tornado sensitivity diagram representing change in pneumococcal death estimates in children <5 years of age in the pre-vaccine era, when values of key variables are modified. (EPS)
