# Supplementary material for: Estimated severe pneumococcal disease cases and deaths before and after pneumococcal conjugate vaccine introduction in children younger than 5 years of age in South Africa
Source: PLoS One. 2017 Jul 3;12(7):e0179905. doi: 10.1371/journal.pone.0179905 (PMC5495214; doi:10.1371/journal.pone.0179905)
Supplement: S1 Text — (DOCX) [file pone.0179905.s001.docx]

**Estimated Severe Pneumococcal Disease Cases and Deaths before and after Pneumococcal Conjugate Vaccine Introduction in Children Younger than 5 Years of Age in South Africa**

Claire von Mollendorf, Stefano Tempia, Anne von Gottberg, Susan Meiring, Vanessa Quan, Charles Feldman, Jeane Cloete, Shabir A. Madhi, Katherine L. O’Brien, Keith P. Klugman, Cynthia G. Whitney, Cheryl Cohen

**Methods**

**Model overview**

**Estimated pneumococcal cases, deaths and incidence rates by clinical syndrome in the pre-pneumococcal conjugate vaccine (PCV) period (2005-2008) and post-PCV period (2012-2013)**

A number of steps were followed to estimate pneumococcal case numbers, numbers of deaths and incidence rates in both the pre- and post-PCV periods:

**STEP 1** (S1 Fig.)

1. All GERMS-SA hospitalised IPD cases from both enhanced and non-enhanced sites from 2005 to 2008 and 2012 to 2013, were included in the baseline numbers on which the model was based because specimen type was available for all cases. An average of the case numbers for the pre-vaccine period (2005-2008) and a period post-vaccine introduction (2012-2013) was calculated. Based on specimen type (i.e. CSF or non-CSF), IPD cases were defined as meningitis or other cases. Cases numbers were determined by province and age group (<1 and 1-4 years) for each of the time periods (2005-2008 and 2012-2013).
2. Pneumococcal meningitis cases were defined as all IPD cases with *S. pneumoniae* isolated from CSF (enhanced and non-enhanced sites) or IPD cases where the organism was isolated from blood and the clinical diagnosis was meningitis (enhanced sites). The proportion of meningitis cases diagnosed clinically, with only a positive blood culture, was determined from enhanced site cases and extrapolated to non-enhanced site cases (as for other syndromes). This extrapolation was done by province and age for each of the time periods (2005-2008 and 2012-2013). The calculated non-enhanced site cases were combined with the diagnosed meningitis cases by province and age group (<1 year and 1-4 years).
3. Non-meningitis pneumococcal cases were defined as all the remaining IPD cases that were not meningitis cases. Among the remaining IPD cases we applied the proportions of bacteraemic pneumococcal pneumonia and non-pneumonia non-meningitis IPD cases observed at enhanced sites in each year and by province and age group (<1 and 1-4 years) to the non-meningitis IPD cases at non-enhanced sites to calculate case numbers by syndrome. Case counts, by syndrome, from non-enhanced sites were combined with those from enhanced sites for each of the categories.

**STEP 2 (**S2 Fig.)

1. Pneumococcal meningitis cases were adjusted for specimen-taking practices by province (see bullet 5b) as certain provinces had better access to care and more cerebrospinal fluid specimens were taken in certain provinces compared with others, assuming that IPD incidence was similar across all provinces. We totalled meningitis cases and stratified by HIV (see bullet 9) to calculate numbers of HIV-infected and –uninfected meningitis pneumococcal cases.
2. For bacteraemic pneumococcal pneumonia we adjusted for incomplete blood culturing in the Gauteng Province (see bullet 5a) and for specimen-taking practices by province (see bullet 5b) as certain provinces had better access to care and some provinces took more blood culture specimens for patients presenting with pneumonia. We totalled bacteraemic pneumococcal pneumonia cases and stratified by HIV (see bullet 9) to calculate numbers of HIV-infected and –uninfected bacteraemic pneumococcal pneumonia cases.
   1. We assumed that Gauteng Province had the highest rate of specimen-taking for pneumonia but knew that not all children with pneumonia would have had a blood culture taken as part of routine practice. In contrast in a clinical trial setting all children presenting with pneumonia had a blood culture taken. To account for incomplete blood culture collection among children hospitalised with pneumonia, we adjusted the measured rate of bacteraemic pneumococcal pneumonia in 2005-2008 by a ratio (23:1) comparing the bacteraemic pneumococcal pneumonia hospitalisation incidence from a PCV clinical trial (control arm) in Soweto [1, 2] to the measured bacteraemic pneumococcal pneumonia incidence in the same province (2005-2008). The same ratio was used to adjust the 2012-2013 measured case numbers.
   2. Specimen-taking practices varied across facilities and provinces. To account for differences in specimen-taking practices and the underdiagnosis of IPD, we adjusted GERMS case numbers for meningitis, bacteraemic pneumonia and non-pneumonia non-meningitis using a specimen ratio where the Gauteng province incidence was estimated to be the baseline (= 1,0) and have the most complete specimen taking after been adjusted (as in bullet 5a), compared with the incidence rates in other provinces by year. Data on total numbers of blood and cerebrospinal fluid specimens taken on an annual basis and submitted to public-sector laboratories was obtained from the National Health Laboratory Service Corporate Data Warehouse. These data were broken down by province, age group and year. We assumed that this adjustment would account for children who did not reach a hospital for care as this also differed between provinces.
3. To calculate the number of hospitalised non-bacteraemic pneumococcal pneumonia cases we used published data on the PCV9 attributable reduction (VAR) ratio of clinical pneumonia to all bacteraemic pneumococcal pneumonia [2]. We divided the VAR for clinical pneumonia (410 cases/100,000 child-years) by the VAR for all bacteraemic pneumococcal pneumonia (37 cases/100,000 child-years) to obtain a ratio of 11:1 which takes into account differences in vaccine efficacy and incidence between the non-bacteraemic and bacteraemic pneumonia groups. We used the clinical pneumonia outcome because it was found to be the most sensitive measure of pneumococcal pneumonia burden in the paediatric clinical trial (i.e. had the highest VAR), however clinical pneumonia has limitations in detection. The total non-bacteraemic pneumococcal pneumonia cases were stratified by HIV (see bullet 9) to calculate numbers of HIV-infected and –uninfected non-bacteraemic pneumococcal pneumonia cases. The relative risk for bacteraemic pneumonia was used for non-bacteraemic pneumonia.
4. For non-pneumonia non-meningitis IPD case numbers we adjusted the observed cases from GERMS for specimen-taking practices by province (see bullet 5b) as certain provinces had better access to care and some provinces took proportionally more blood culture specimens. We totalled the adjusted non-pneumonia non-meningitis IPD cases and stratified by HIV (see bullet 9) to calculate numbers of HIV-infected and –uninfected non-pneumonia non-meningitis IPD cases.
5. To calculate the relative risk (RR) of pneumococcal disease in HIV-infected (HI) versus HIV-uninfected (HU) cases for each syndrome we used the following formula:

$$RR = \frac{HI cases per{year}/{HI population denominator}}{{HU cases per year}/{HU population denominator}}$$

The RR for pneumococcal disease due to HIV infection was estimated by dividing the rate of IPD HIV-infected cases per year to the rate of IPD HIV-uninfected cases per year for each syndrome. The number of HIV-infected cases were calculated by multiplying the prevalence of HIV by the number of enhanced site cases, while HIV-uninfected case numbers were the difference between total and HIV-infected case numbers.

1. We stratified by HIV for all pneumococcal syndromes by estimating the number of HIV-infected (HI) cases in each group using the following formula:

$$HI IPD cases = \frac{1}{\left( \frac{PopHIV-}{RR*PopHIV+} +1 \right) * \frac{1}{IPDTotal}}$$

PopHIV+ and PopHIV- are the age- and year-specific number of HIV-positive and HIV-negative individuals in the population, RR is the age- and year-specific relative risk for pneumococcal hospitalizations due to HIV infection calculated as described in bullet 8 and IPDTotal is the age- and year-specific national number of pneumococcal hospitalizations. We used the HIV RR instead of the HIV prevalence among pneumococcal cases in 2008 because of the changing HIV prevalence in the population from 2005 to 2008 [3].

We calculated the number of HIV-uninfected cases as the difference between the total number of cases and the HIV-infected cases (IPD HIV-uninfected cases = IPD Total cases – IPD HIV-infected cases).

1. We calculated the adjusted number of pneumococcal deaths for each syndrome by age group by multiplying adjusted case number estimates (as calculated above) by case fatality ratios (CFRs) observed at the GERMS-SA enhanced sites for bacteraemic cases (meningitis, bacteraemic pneumonia and non-pneumonia non-meningitis). For non-bacteraemic pneumococcal pneumonia we used CFRs from a study in Kenya [4] that observed case fatality rates among children under 5 years of age admitted to hospital for any reason with and without bacteraemia (all children had blood cultures obtained regardless of their admission diagnosis). The observed CFR for non-bacteraemic admissions was (5.7%) which was 5-fold lower than that observed for bacteraemic admissions (28.2%).
2. For all syndromes we calculated incidence and mortality rates using the adjusted case and death estimates from the model in combination with the and mid-year population estimates obtained from Statistics South Africa as denominators for different age groups (http://www.statssa.gov.za/). The Thembisa model, which accounted for PMTCT and HIV treatment impact, was used for HIV specific denominators [5]. We calculated incidence rate ratios for all syndromes comparing incidence or mortality rates in HIV-infected to HIV-uninfected children (S1 Table).
3. The proportional reduction in different syndromes was based on actual numbers in 2012-2013 and calculated reductions therefore differed by syndrome, age group and HIV status.

**Sensitivity analysis**

A one-way sensitivity analysis was performed by changing one variable at a time to see the effect on the total number of cases and deaths (Tornado diagrams, S3 and S4 Figs).

Parameters that we varied in the sensitivity analysis of cases and deaths included the proportion of under 5 year old deaths that occur in the community, an adjustment for the likelihood of obtaining a blood culture among hospitalised children with suspected pneumococcal disease and the ratio of bacteraemic to non-bacteraemic pneumococcal pneumonia. For estimates of pneumococcal deaths we also assessed the effect of changes in CFRs (see Table 2 in main manuscript for parameters).

1. For the proportion of all deaths that occur in the community (which we did not include in our main analysis) we used vital statistics data from Statistics South Africa which enumerates deaths in the community and in hospitals by syndrome. We calculated the additional proportion of cases that we would expect to have died in the community based on our hospital data and added these cases to the model. The limitation of this assumption is that cases that die in and out of hospital may not be directly comparable with each other in terms of severity and causation.
2. For incomplete blood culturing practices among children hospitalised with IPD, we adjusted the measured rate of bacteraemic pneumococcal pneumonia by a ratio of 8:1 which compared the IPD hospitalisation incidence from the PCV clinical trial (control arm) in Soweto [2] to the measured IPD incidence in the same province.
3. For the ratio of bacteraemic to non-bacteraemic pneumococcal pneumonia we used the same published data on the vaccine attributable reduction (VAR) ratio (11:1) of clinical pneumonia to bacteraemic pneumococcal pneumonia as in our main analysis [2]. Based on a clinical trial in the elderly [6] which incorporated urine antigen testing for non-bacteraemic pneumonia, we tried to account for the apparent underestimation of VE in the paediatric clinical trials. The ratio (1.89) of the VEs from the two clinical was used to inflate the non-bacteraemic pneumococcal pneumonia case numbers [6] calculated in our main analysis.
4. A second VAR calculation using the ratio of CXR-confirmed pneumonia to bacteraemic pneumococcal pneumonia (4:1) [2] and including the additional factor (1.89) accounting for the change in vaccine efficacy [6] was conducted (7.6:1).
5. An alternative calculation of non-bacteraemic pneumococcal pneumonia calculated the number of HIV-infected and –uninfected cases separately and then summed these numbers. The sensitivity of blood culture for diagnosing pneumococcal pneumonia was assumed to be 3-5% in HIV-uninfected children and 18% in HIV-infected children. We also used separate VARs for HIV-infected (5:1) and –uninfected children (38:1) for the ratio of clinical pneumonia to bacteraemic pneumonia from the Soweto pneumococcal clinical trial [2].
6. For the CFR ratio of bacteraemic to non-bacteraemic pneumococcal pneumonia we used the death risk ratio reported by a trial from The Gambia [7]. This ratio (3:1) was considered an underestimate as it compared the adjusted risk ratio for death of end-point pneumonia (1.98) which included the highest proportion of bacteraemic pneumonia to the adjusted risk ratio for death of ‘other infiltrates /abnormalities’ pneumonia (0.66) which had the lowest proportion of positive blood cultures. [8]
7. Lastly, we included a second study to calculate the CFR ratio of bacteraemic to non-bacteraemic pneumococcal pneumonia. For non-bacteraemic pneumococcal pneumonia we used the ratio of CFR (5:1) among all-cause community-acquired bacteraemic (28.2%) to all-cause non-bacteraemic hospital admissions (5.7%) reported in the literature from Kenya [4, 9]

**Results**

**Impact of the pneumococcal conjugate vaccine and other interventions on the burden of disease**

To crudely estimate the relative contribution of PCV and HIV-related interventions we assumed similar disease rates between 2005-2008 and 2012-2013 and used data on the impact of HIV interventions in HIV-infected children from a previous impact study (31% reduction) [3]. This translated into a total of approximately 37,223 cases potentially averted by the vaccine in children <5 years of age in 2012-2013, 12,819 in HIV-infected and 24,405 in HIV-uninfected children. In the same age group 1146 deaths were assumed averted, 390 in HIV-infected and 757 in HIV-uninfected children.

**Sensitivity analysis**

S2 and S3 Tables demonstrate the key variables altered in the sensitivity analysis for 2005-2008 and 2012-2013 for number of cases and number of deaths respectively.

The highest mortality rates in children <5 years of age was a death rate of 230 per 100,000 py in 2005-2008 and 62 per 100,000 py in 2013.

**Discussion**

The greatest burden of pneumococcal disease in this study presented with pneumonia, at a 96-fold higher rate than meningitis. This was similar to rates described in previous studies where pneumonia made up the bulk of pneumococcal disease (90-fold higher than meningitis) [10]. Even though there has been a reduction in pneumococcal numbers, disease burden as well as pneumococcal death rates still remains significant in the post-PCV era.

The pre-PCV pneumococcal incidence rates calculated by this model were comparable to those from a clinical trial conducted in South Africa in the pre-antiretroviral treatment, pre-vaccine era from 1998-2001. The clinical trial reported an IPD incidence of 331 per 100,000 py in young children in the placebo arm [1] which was slightly higher than this burden model rate of 277 per 100,000 py (2005-2008). The same trial demonstrated a bacteraemic pneumococcal pneumonia incidence for all children of 196 per 100,000 py [2] which was similar to our estimates of 163 per 100,000 py. The non-bacteraemic pneumococcal pneumonia rates (1,797 per 100,000 py) much lower than the observed incidence of clinical lower respiratory tract infection (3,565 per 100,000) rates among the placebo arm of the clinical trial [2]. Our model utilised parameters from this clinical trial to calculate the burden of non-bacteraemic from bacteraemic pneumonia, and this may have contributed to similar rates.

Although we did not calculate costs of pneumococcal hospitalizations, there have been studies from Latin America which documented the substantial cost of pneumococcal disease and found that PCV introduction was cost-saving [11, 12]. With approximately 26,000 hospitalised cases averted annually in South Africa in 2013, it is expected that PCV will have significant cost reductions for the health system despite the expense of the vaccine. A health economic study in South Africa [13] calculated the median total cost for a severe acute respiratory infection (SARI) hospitalization as ZAR 8804.25; assuming a similar cost for pneumococcal disease, ZAR 1,133,987,400 (US$ 80,424,638; exchange rate ZAR:US$ 14:1 [14]) could be saved by 2013 in direct hospitalization costs compared with those that would occur without the PCV program.

**Figures**

**S1 Fig.: Initial step in estimating the burden of invasive and non-invasive pneumococcal cases in children aged <5 years in South Africa, 2005-2008 and 2012-2013**

ES = enhanced sites, NES = non-enhanced sites, IPD = invasive pneumococcal disease

*Includes cases positive for pneumococcus from CSF at ES and NES, and among ES sites, cases with pneumococcus from blood culture along with a clinical meningitis diagnosis

**S2 Fig.: Second step in estimating the burden of invasive and non-invasive pneumococcal cases in children <5 years in South Africa, 2005-2008 and 2012-2013**

**S3 Fig.: Tornado sensitivity diagram representing change in pneumococcal case estimates in children <5 years of age in the pre-vaccine era, when values of key variables are modified.**

Footnote: *• Community death rates = deaths in the community in children <5 years derived from Statistics South Africa vital statistics data. • Vaccine probe estimates = Incomplete blood culturing practice adjustment derived from ratio of IPD hospitalisation incidence from PCV clinical trial in Soweto to measured GERMS IPD incidence (8:1). • Additional adjustment of 1.89 = Adjustment to account for underestimation of VE from clinical trials.*

*• Ratio of NBP to BPP cases = VAR ratio of bacteraemic to CXR-confirmed non-bacteraemic pneumococcal pneumonia (1: 7.6). • Separate HIV NBP calculation = Alternative calculation of non-bacteraemic pneumococcal pneumonia separately for HIV-infected and –uninfected children using separate VARs.*

**S4 Fig.: Tornado sensitivity diagram representing change in pneumococcal death estimates in children <5 years of age in the pre-vaccine era, when values of key variables are modified.**

Footnote: *• Community death rates = deaths in the community in children <5 years derived from Statistics South Africa vital statistics data. • Vaccine probe estimates = Incomplete blood culturing practice adjustment derived from ratio of IPD hospitalisation incidence from PCV clinical trial in Soweto to measured GERMS IPD incidence (8:1). • Additional adjustment of 1.89 = Adjustment to account for underestimation of VE from clinical trials.*

*• Ratio of NBP to BPP cases = VAR ratio of bacteraemic to CXR-confirmed non-bacteraemic pneumococcal pneumonia (1: 7.6). • Separate HIV NBP calculation = Alternative calculation of non-bacteraemic pneumococcal pneumonia separately for HIV-infected and –uninfected children using separate VARs. • Adjusted CFR for NBP/BPP Gambia (3:1): Adjusted death risk ratio of end-point pneumonia to ‘other infiltrates /abnormalities’ pneumonia (1.98:0.66=3:1). • Adjusted CFR for NBP/BPP Kenya (5:1): Ratio of CFR (5:1) among all-cause community-acquired bacteraemic (28.2%) to all-cause non-bacteraemic hospital admissions (5.7%).*

**References**

1. Klugman KP, Madhi SA, Huebner RE, Kohberger R, Mbelle N, Pierce N. A trial of a 9-valent pneumococcal conjugate vaccine in children with and those without HIV infection. The New England journal of medicine. 2003;349(14):1341-8. doi: 10.1056/NEJMoa035060 [doi];349/14/1341 [pii].

2. Madhi SA, Kuwanda L, Cutland C, Klugman KP. The impact of a 9-valent pneumococcal conjugate vaccine on the public health burden of pneumonia in HIV-infected and -uninfected children. Clinical infectious diseases : an official publication of the Infectious Diseases Society of America. 2005;40(10):1511-8. doi: CID35328 [pii];10.1086/429828 [doi].

3. von Gottberg A, de Gouveia L, Tempia S, Quan V, Meiring S, von Mollendorf C, et al. Effects of vaccination on invasive pneumococcal disease in South Africa. The New England journal of medicine. 2014;371(20):1889-99. doi: 10.1056/NEJMoa1401914. PubMed PMID: 25386897.

4. Berkley JA, Lowe BS, Mwangi I, Williams T, Bauni E, Mwarumba S, et al. Bacteremia among children admitted to a rural hospital in Kenya. The New England journal of medicine. 2005;352(1):39-47. doi: 10.1056/NEJMoa040275. PubMed PMID: 15635111.

5. Johnson LF, Chiu C, Myer L, Davies MA, Dorrington RE, Bekker LG, et al. Prospects for HIV control in South Africa: a model-based analysis. Global health action. 2016;9:30314. doi: 10.3402/gha.v9.30314. PubMed PMID: 27282146; PubMed Central PMCID: PMC4901512.

6. Bonten MJ, Huijts SM, Bolkenbaas M, Webber C, Patterson S, Gault S, et al. Polysaccharide conjugate vaccine against pneumococcal pneumonia in adults. The New England journal of medicine. 2015;372(12):1114-25. doi: 10.1056/NEJMoa1408544. PubMed PMID: 25785969.

7. Enwere G, Cheung YB, Zaman SM, Akano A, Oluwalana C, Brown O, et al. Epidemiology and clinical features of pneumonia according to radiographic findings in Gambian children. Tropical medicine & international health : TM & IH. 2007;12(11):1377-85. doi: 10.1111/j.1365-3156.2007.01922.x. PubMed PMID: 18045264.

8. Forgie IM, O'Neill KP, Lloyd-Evans N, Leinonen M, Campbell H, Whittle HC, et al. Etiology of acute lower respiratory tract infections in Gambian children: I. Acute lower respiratory tract infections in infants presenting at the hospital. The Pediatric infectious disease journal. 1991;10(1):33-41. PubMed PMID: 1848364.

9. Ayieko P, Griffiths UK, Ndiritu M, Moisi J, Mugoya IK, Kamau T, et al. Assessment of health benefits and cost-effectiveness of 10-valent and 13-valent pneumococcal conjugate vaccination in Kenyan children. PLoS One. 2013;8(6):e67324. doi: 10.1371/journal.pone.0067324. PubMed PMID: 23826268; PubMed Central PMCID: PMC3691111.

10. O'Brien KL, Wolfson LJ, Watt JP, Henkle E, Deloria-Knoll M, McCall N, et al. Burden of disease caused by *Streptococcus pneumoniae* in children younger than 5 years: global estimates. Lancet. 2009;374(9693):893-902. doi: S0140-6736(09)61204-6 [pii];10.1016/S0140-6736(09)61204-6 [doi].

11. Constenla D. Evaluating the costs of pneumococcal disease in selected Latin American countries. Revista panamericana de salud publica = Pan American journal of public health. 2007;22(4):268-78. PubMed PMID: 18078589.

12. Bahia L, Toscano CM, Takemoto ML, Araujo DV. Systematic review of pneumococcal disease costs and productivity loss studies in Latin America and the Caribbean. Vaccine. 2013;31 Suppl 3:C33-44. doi: 10.1016/j.vaccine.2013.05.030. PubMed PMID: 23777689.

13. Cohen AL. The Burden and Cost of Influenza-Associated Illness in South Africa–Preliminary Results. WHO Consultation on National, Regional and Global Estimates of the Burden of Influenza Disease; July 2016; Geneva, Switzerland.

14. Bloomberg Finance L.P. Bloomberg Markets 2016. Available from: <http://www.bloomberg.com/quote/USDZAR:CUR>. Accessed 07 September 2016.
