# Supplementary material for: Estimated severe pneumococcal disease cases and deaths before and after pneumococcal conjugate vaccine introduction in children younger than 5 years of age in South Africa
Source: PLoS One. 2017 Jul 3;12(7):e0179905. doi: 10.1371/journal.pone.0179905 (PMC5495214; doi:10.1371/journal.pone.0179905)
Supplement: S1 Table — (DOCX) [file pone.0179905.s002.docx]

**S1 Table: Population denominators from the Thembisa model for children <5 years of age in South Africa, 2005-2008 and 2012-2013**

|  | **2005** | **2006** | **2007** | **2008** | **2005_2008** | **2012** | **2013** | **2012_2013** |
| --- | --- | --- | --- | --- | --- | --- | --- | --- |
|  | **<1 year** | | | | | | | |
| **Pop Total** | 1098294 | 1133907 | 1163590 | 1173199 | 1142247 | 1191044 | 1189106 | 1190075 |
| **Pop HIV+** | 42915 | 39989 | 35587 | 28069 | 36640 | 11318 | 9776 | 10547 |
| **Pop HIV-** | 1055379 | 1093918 | 1128003 | 1145131 | 1105608 | 1179726 | 1179330 | 1179528 |
|  | **1-4 years** | | | | | | | |
| **Pop Total** | 3841788 | 3978612 | 4160544 | 4340409 | 4080338 | 4550033 | 4572477 | 4561255 |
| **Pop HIV+** | 184941 | 184493 | 180935 | 173382 | 180938 | 98439 | 84020 | 91230 |
| **Pop HIV-** | 3656847 | 3794119 | 3979609 | 4167028 | 3899401 | 4451593 | 4488457 | 4470025 |
|  | **<5 year** | | | | | | | |
| **Pop Total** | 4940082 | 5112520 | 5324133 | 5513608 | 5222586 | 5741077 | 5761583 | 5751330 |
| **Pop HIV+** | 227856 | 224482 | 216522 | 201450 | 217578 | 109757 | 93796 | 101777 |
| **Pop HIV-** | 4712226 | 4888037 | 5107611 | 5312158 | 5005008 | 5631320 | 5667787 | 5649553 |
