# Supplementary material for: Estimated severe pneumococcal disease cases and deaths before and after pneumococcal conjugate vaccine introduction in children younger than 5 years of age in South Africa
Source: PLoS One. 2017 Jul 3;12(7):e0179905. doi: 10.1371/journal.pone.0179905 (PMC5495214; doi:10.1371/journal.pone.0179905)
Supplement: S2 Table — (DOCX) [file pone.0179905.s003.docx]

**Table S2. Sensitivity analysis for case numbers showing key variables altered in analysis, 2005-2008 and 2012-2013**

| **Key variables altered in analysis** | **Number of cases** | |
| --- | --- | --- |
|  | **2005-2008** | **2012-2013** |
| 1. Base numbers | 107,600 | 41,700 |
| 1. Community death rates | 109,500 | 48,800 |
| 1. Altered blood culturing estimates | 98,100 | 34,400 |
| 1. NBP/BPP VAR ratio of 11:1 with 1.89 adjustment | 196,100 | 78,100 |
| 1. NBP/BPP VAR ratio of 7.6:1 | 80,300 | 26,500 |
| 1. Altered NBP calculations with separate HIV estimates | 144,900 | 59,500 |

NBP = non-bacteraemic pneumococcal pneumonia; BPP = bacteraemic pneumococcal pneumonia
