# Supplementary material for: Estimated severe pneumococcal disease cases and deaths before and after pneumococcal conjugate vaccine introduction in children younger than 5 years of age in South Africa
Source: PLoS One. 2017 Jul 3;12(7):e0179905. doi: 10.1371/journal.pone.0179905 (PMC5495214; doi:10.1371/journal.pone.0179905)
Supplement: S3 Table — (DOCX) [file pone.0179905.s004.docx]

**Table S3. Sensitivity analysis for numbers of deaths showing key variables altered in analysis, 2005-2008 and 2013**

| **Key variables altered in analysis** | **Number of deaths** | |
| --- | --- | --- |
|  | **2005-2008** | **2012-2013** |
| 1. Base numbers | 5000 | 1900 |
| 1. Community death rates | 3700 | 1600 |
| 1. Altered blood culturing estimates | 2500 | 850 |
| 1. NBP/BPP VAR ratio of 11:1 with 1.89 adjustment | 4500 | 1700 |
| 1. NBP/BPP VAR ratio of 7.6:1 | 3400 | 1200 |
| 1. Altered NBP calculations with separate HIV estimates | 11900 | 1500 |
| 1. Adjusted case fatality ratio for BPP to NBP from Gambia study (3:1 ratio) | 7600 | 3300 |
| 1. Adjusted case fatality ratio for BPP to NBP from Kenya study (5:1 ratio) | 6900 | 2100 |

NBP = non-bacteraemic pneumococcal pneumonia; BPP = bacteraemic pneumococcal pneumonia
